# Supplementary material for: Advancing macromolecular structure determination with microsecond X-ray pulses at a 4th generation synchrotron
Source: Commun Chem. 2025 Jan 7;8:6. doi: 10.1038/s42004-024-01404-y (PMC11707155; doi:10.1038/s42004-024-01404-y)
Supplement: Supplementary file 2 — Supplementary Material [file 42004_2024_1404_MOESM2_ESM.pdf]

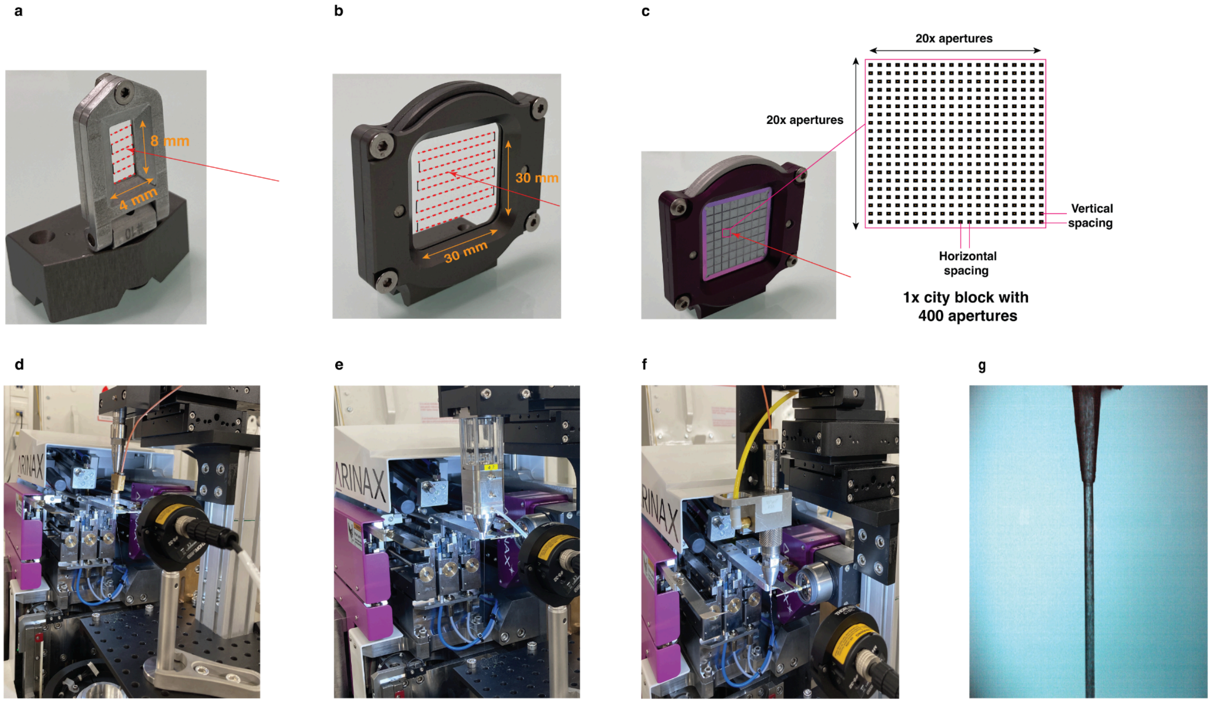

Supplementary Fig. 1 | Fixed-Target and HVE SµX experiments. a-b. Schematic representation of S-shaped data collection trajectories on small and large foils respectively. Horizontal spacing between pulses and vertical spacing, between lines, are defined in the user interface. **c**. Oxford-chip mounted on a large foil frame. The inset illustrates a single city-block structured in 20 x 20 apertures which are equally spaced in horizontal and vertical directions. **d–f**. ASU-HVE, MPI-HVE, SACLA-HVE mounted on MD3upSSX diffractometer at ID29. **g**. A stable extruded matrix Hydroxyethyl cellulose embedding thaumatin crystals.

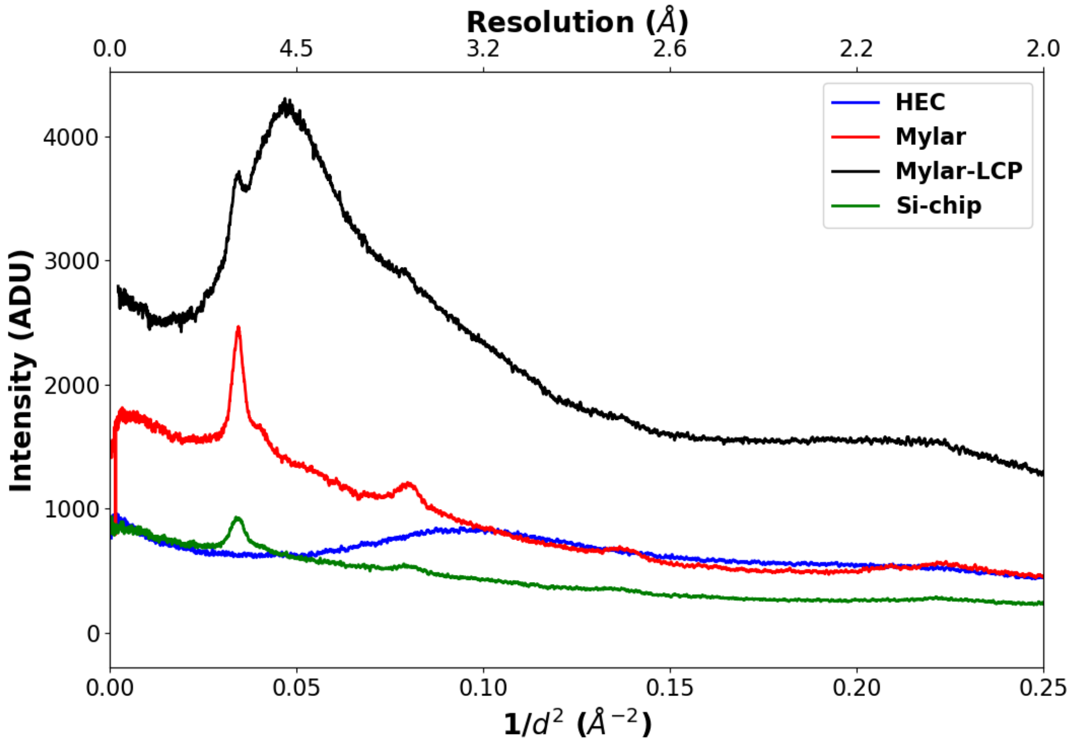

Supplementary Fig. 2 | Background comparison of HVE media and fixed target foils. Azimuthal integration of single frames (containing no diffraction) collected with different sample supports and media: Si-chip (enclosed between two mylar films) in green, small foils with 26 µm Mylar and mother liquor in red, small foils with 26 µm Mylar with LCP media, in black. Integration of frames collected with HVE and HEC matrix in blue. 1 photon corresponds to 478 Analog-Digital-Units (ADUs).

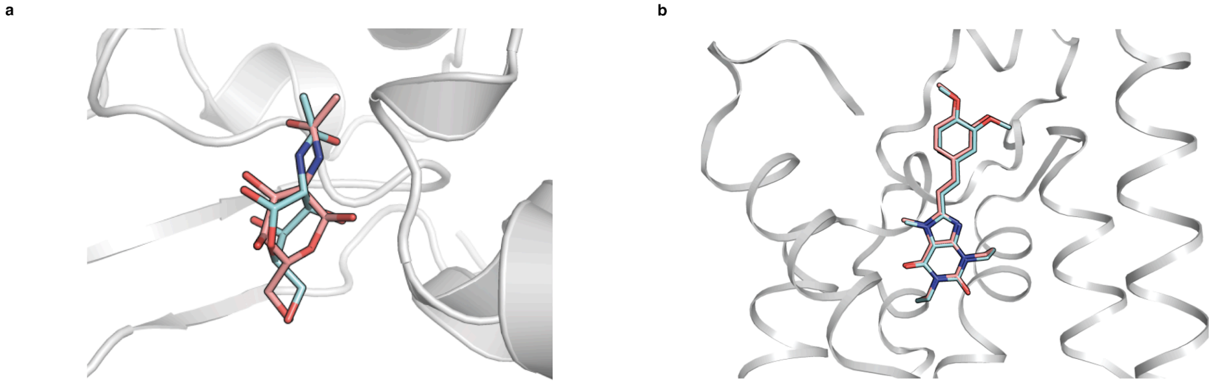

Supplementary Fig. 3 | Overlay of refined vs phenix.ligand docked structure. a. Lysozyme active site with GlcNAc ligand automatically placed by *phenix.ligandfit* (cyan), performed using only 2000 merged frames, overlaid with the refined position (salmon), using the full dataset. The figure shows a correct placement with a 180° rotation along the glucose C2-C5 direction. **b**. $A_{2A}R$ ligand site with Istradefylline automatically placed by *phenix.ligandfit* (cyan), using only 2000 merged frames, overlaid with the refined position (salmon), using the full dataset.

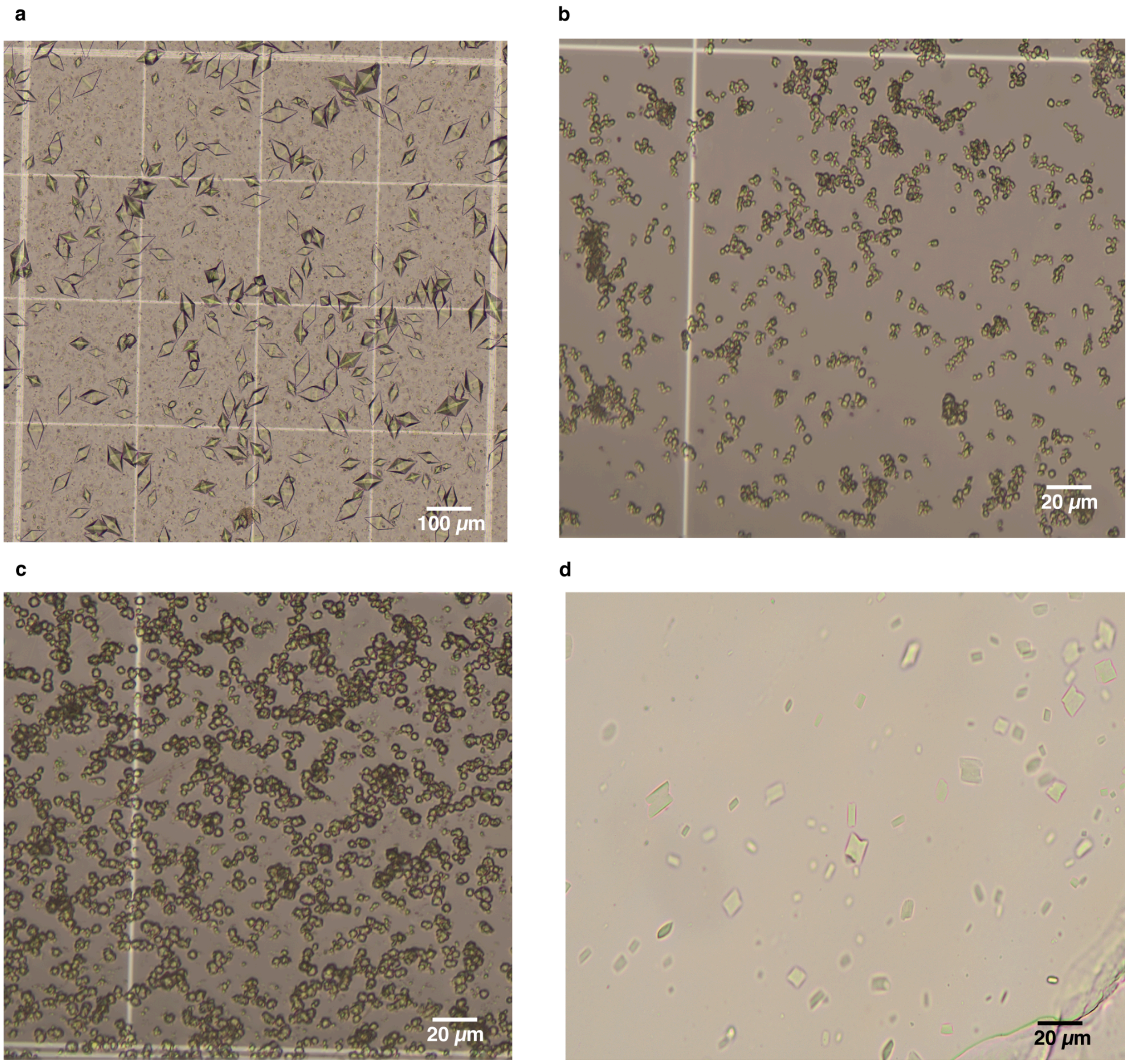

Supplementary Fig. 4 | Microscope images for microcrystals of Thaumatin, Lysozyme, Proteinase-K and $A_{2A}R$ used in the experiments, are shown in panel **(a–d)** respectively. Scales bar provided by Olympus cellSens software according to the magnification.

Supplementary Table 1 | Summary of microcrystals' motion during data collection. Values are calculated from the motor scanning speed for fixed target and from elution speed for HVE

Spacing between two X-ray pulses (µm)	Translational speed (µm/ms)	Crystal movement within a single 90-µs X-ray pulse (µm)
Crystal in Fixed-target		
10	2.3	0.2
15	3.5	0.3
20	4.6	0.4
50	11.6	1.0
125	28.9	2.6
Crystal in HVE jet		
	2.0-3.0	0.2-0.3

Supplementary Table 2 | Data processing and refinement statistics for experiments with fixed-target

	HEWL Si-chip		Proteinase-K foils			A_{2A}R-Istradefylline
	apo	GlcNAc	10-µm	20-µm	50-µm	
**PDB Code**	**9FUD**	**9FUE**	**9FTX**	**9FTY**	**9FU1**	**9FUP**
**Data collection**						
# collected images	51,200	51,200	327,600	163,600	250,000	435,708
# hits	29,900	32,610	45,451	34,763	25,719	120,366
# indexed images	6,222	7,234	17,097	10,011	8,088	17,297
# lattices	6,776	7,973	18,299	10,726	8,661	17,773
Space group	$P4_32_12$	$P4_32_12$	$P4_32_12$	$P4_32_12$	$P4_32_12$	$C222_1$
Unit-Cell						
a, b, c (Å)	78.70, 78.70, 37.86	78.56, 78.56, 37.79	68.72, 68.72, 108.97	68.72, 68.72, 108.97	68.69, 68.69, 108.93	40.17, 179.40, 142.34
$\alpha=\beta=\gamma$ (°)	90	90	90	90	90	90
Resolution (Å)	55.6–2.05 (2.08–2.05)	55.6–2.05 (2.08–2.05)	58.1–2.00 (2.03–2.00)	58.1–2.00 (2.03–2.00)	58.1–2.00 (2.03–2.00)	89.7–2.50 (2.54–2.50)
# unique reflections	7,923	7,877	18,372	18,372	18,350	18,404
$\langle I/\sigma(I)\rangle$	4.16 (0.36)	4.64 (0.40)	5.24 (1.02)	3.94 (0.90)	3.33 (0.64)	4.33 (0.62)
Completeness (%)	100 (100)	100 (100)	100 (100)	100 (100)	100 (99.89)	99.94 (100)
Multiplicity	101.0 (36.3)	123.5 (44.8)	221.4 (61.8)	133.2 (38.0)	99.1 (27.9)	208.6 (146.0)
CC* (%)	99.58 (36.95)	99.63 (45.07)	98.40 (81.73)	96.53 (74.65)	96.51 (49.48)	98.61 (52.44)
Rsplit (%)	16.30 (256.95)	14.37 (249.36)	21.75 (104.68)	32.07 (128.12)	31.79 (175.95)	23.11 (246.47)
Wilson B value (Å^2)	42.02	40.66	19.51	17.59	22.68	56.92
Average dose / crystal (kGy)†	870	870	851	851	851	135
**Structure Refinement**						
Resolution range	55.6–2.05 (2.09–2.05)	55.6–2.05 (2.09–2.05)	58.1–2.00 (2.03–2.00)	58.1–2.00 (2.03–2.00)	58.1–2.00 (2.03–2.00)	55.8–2.50 (2.54–2.50)
# reflections	7,787 (323)	7,822 (365)	18,304 (890)	18,253 (889)	18,288 (896)	17,921 (796)
R-work (%)	21.46 (37.34)	20.67 (35.32)	21.84 (32.80)	24.04 (34.98)	18.22 (32.10)	25.08 (36.12)
R-free (%)	25.03 (39.05)	26.50 (42.23)	26.24 (33.83)	26.24 (35.56)	22.38 (35.43)	28.77 (47.30)
# non-hydrogen atoms						
Protein	977	985	1998	1989	1998	2816
Ligand	N/A	15	4	4	4	346
Ion	5	5	2	2	2	1
Solvent	36	41	189	173	176	42
Average B-factors (Å^2)						
Protein	48.08	47.47	17.29	15.24	21.83	80.90
Ligand	NA	56.46	40.96	28.80	39.84	79.55
Ion	55.67	63.08	19.93	17.52	23.81	56.86
Solvent	48.88	51.60	25.91	22.84	31.73	64.36
Ramachandran (%)						
Favored (%)	96.85	96.85	96.75	96.39	96.39	98.42
	3.15	3.15	3.25	3.61	3.61	1.58
Allowed (%)						
Outliers (%)	0	0	0	0	0	0
R.m.s. deviations						
Bond lengths (Å)	0.003	0.002	0.002	0.002	0.003	0.002
Bond angles (°)	0.500	0.490	0.460	0.440	0.570	0.560

†Average dose (exposed region) [ADER]

Supplementary Table 3 | Data processing and refinement statistics for experiments with various High-viscosity extruders (HVEs)

	Thaumatin		
	**HVE-ASU**	**HVE-SACLA**	**HVE-MPI**
**PDB Code**	**9FTU**	**9FTS**	**9FTV**
**Data collection**			
# collected images	500,000	500,000	500,000
# hits	194,027	115,869	195,091
# indexed images	114,200	58,334	138,418
# lattices	122,076	60,223	151,265
Space group	$P4_12_12$	$P4_12_12$	$P4_12_12$
Unit-Cell			
a=b, c (Å)	58.55, 151.49	58.48, 151.35	58.49, 151.34
$\alpha=\beta=\gamma$ (°)	90	90	90
Resolution (Å)	54.6–1.75 (1.78–1.75)	54.6–1.75 (1.78–1.75)	54.6–1.75 (1.78–1.75)
# unique reflections	27,591	27,498	27,502
$\langle I/\sigma(I)\rangle$	18.82 (1.51)	9.29 (0.47)	22.25 (2.06)
Completeness (%)	100 (100)	100 (100)	100 (100)
Multiplicity	1,420.1 (87.4)	595.4 (37.6)	2,001.4 (126.8)
CC* (%)	99.87 (91.00)	99.58 (56.98)	99.93 (95.08)
Rsplit (%)	5.14 (50.28)	10.83 (177.36)	4.23 (38.92)
Wilson B value ($Å^2$)	23.39	23.76	24.28
Average dose / crystal (kGy)[†§]	228-230	228-230	228-230
**Structure Refinement**			
Resolution range	46.3–1.75 (1.78–1.75)	46.3–1.75 (1.78–1.75)	46.3–1.75 (1.78–1.75)
# reflections	27,505 (1,349)	27,333 (1,269)	27,409 (1,339)
R-work (%)	13.80 (25.33)	15.17 (38.68)	14.22 (25.68)
R-free (%)	16.67 (31.04)	18.17 (41.00)	17.17 (26.78)
# non-hydrogen atoms			
Protein	1,541	1,544	1,548
Ligand	10	10	10
Ion	N/A	N/A	N/A
Solvent	192	164	179
Average B-factors ($Å^2$)			
Protein	22.74	23.85	23.53
Ligand	21.97	22.70	22.00
Ion	N/A	N/A	N/A
Solvent	39.22	37.32	38.48
Ramachandran (%)			
Favored (%)	98.53	99.02	99.02
Allowed (%)	1.47	0.98	0.98
Outliers (%)	0	0	0
R.m.s. deviations			
Bond lengths (Å)	0.013	0.014	0.006
Bond angles (°)	1.240	1.260	0.860

[†]Average dose (exposed region) [ADER]
[§]Dose range due to heterogeneous crystal sizes (12x12x45 µm - 45x45x80 µm)

Supplementary Table 4 | Summary of structure overall statistics for "data titration". The resolution cutoff applied for the different merged datasets are the same as the Lysozyme-GlcNAc and A2AR-Istradefylline models, 2.05 and 2.5 Å respectively.

# Merged	CC* (%)	I/σI	Completeness (%)	Multiplicity	# Unique Reflections	Rwork / Rfree (%)
**HEWL-GlcNAc**						
500	95.28	1.34	94.71	8.09	7,459	27.38 / 31.15
1,000	97.51	1.81	99.31	16.38	7.822	24.77 / 29.42
2,000	98.79	2.46	99.92	33.11	7,870	22.17 / 26.88
5,000	99.49	3.80	100	83.67	7,877	20.68 / 24.47
**A_{2A}R-Istradefylline**						
500	95.39	1.90	60.26	11.12	11,097	31.59 / 36.66
1,000	97.20	1.83	74.05	18.32	13,636	29.89 / 33.04
2,000	98.29	2.12	87.58	30.83	16,128	30.58 / 33.94
5,000	98.23	2.94	98.47	78.31	18,133	26.40 / 28.91

Supplementary Table 5 | Parameters used for the dose calculation with RADDOSE-3D for Thaumatin, Lysozyme, Proteinase-K and $A_{2A}R$

**Sample Delivery Method**	**Injector**	**Chip**	**Foils**	**Foils**
Protein	Thaumatin	Lysozyme	Proteinase-K	$A_{2A}R$
Ring Current (mA)	200	65	200	200
Transmission (%)	15	100	24	24
Normalized flux (ph/sec)	2.40×10^{14}	5.20×10^{14}	3.80×10^{14}	3.80×10^{14}
Pulse length (µs)	90	90	90	90
Beam size (h x v)	4 x 2 µm	4 x 2 µm	4 x 2 µm	4 x 2 µm
Crystal Size (X, Y, Z)	12 x 12 x 45 μm^3 to 45 x 45 x 80 μm^3	5 x 5 x 5 μm^3	4 x 4 x 4 μm^3	10 x 10 x 2 μm^3
Space group	$P4_12_12$	$P4_32_12$	$P4_32_12$	$C222_1$
Unit cell parameters				
a, b, c (Å)	58.5, 58.5, 151.4	78.6, 78.6, 37.8	68.7, 68.7, 109	40.2, 179.4, 142.4
α, β, γ (°)	90, 90, 90	90, 90, 90	90, 90, 90	90, 90, 90
Monomers / unit cell	8	8	8	8
Number of residues / monomer	206	129	279	383
Heavy Atoms (Cys/Met + Ions)	16 Cys, 1 Met	8 Cys, 2 Met, 1 Na, 4 Cl	5 Cys, 5 Met, 2 Ca	14 Cys, 8 Met, 1 Na
Solvent Content	56 %	39 %	44 %	52 %
Protein Buffer	0.1 M HEPES pH 7.0	20 mM sodium acetate pH 4.6	0.02 M MES pH 6.5	25 mM HEPES pH 7.4<br>150 mM NaCl<br>0.03 % (w/v) DDM<br>0.006 % (w/v) CHS<br>10 µM Istradefylline<br>9.9 MAG (monoolein)<br>10 % (w/w) cholesterol
Crystallisation Buffer	0.1 M HEPES pH 7.5<br>1,8 M Na/K tartrate	1 M sodium acetate pH 3.0<br>20 % (w/v) NaCl<br>5 % (w/v) PEG 6000	0.1 M MES pH 6.5<br>0.5 M sodium nitrate<br>0.1 M calcium chloride	0.1 M tri-sodium citrate pH 5.0<br>50 mM sodium thiocyanate<br>29-30 % (v/v) PEG 400<br>1 % (v/v) 1,6-hexanediol
Material type encasing irradiated sample and thickness	N/A	Mylar 13 µm	Mylar 13 µm	Mylar 13 µm
Average dose per crystal (Whole Crystal)	9 - 122 kGy	870 kGy	851 kGy	124 kGy

Average dose per crystal (Exposed Region)	228 - 230 kGy	870 kGy	851 kGy	135 kGy
