# Supplementary material for: Advancing macromolecular structure determination with microsecond X-ray pulses at a 4th generation synchrotron
Source: Commun Chem. 2025 Jan 7;8:6. doi: 10.1038/s42004-024-01404-y (PMC11707155; doi:10.1038/s42004-024-01404-y)
Supplement: Supplementary file 3 — Description of Additional Supplementary Files [file 42004_2024_1404_MOESM3_ESM.pdf]

Description of Additional Supplementary Files

File name- Supplementary Data 1

File description- Dose calculation scripts are provided as Supplementary Data 1 file.
