# Supplementary material for: Advancing macromolecular structure determination with microsecond X-ray pulses at a 4th generation synchrotron
Source: Commun Chem. 2025 Jan 7;8:6. doi: 10.1038/s42004-024-01404-y (PMC11707155; doi:10.1038/s42004-024-01404-y)
Supplement: Supplementary file 4 — Supplementary Data 1 [file 42004_2024_1404_MOESM4_ESM.pdf]

Supplementary Data 1: Dose calculation scripts

Thaumatin

```
#  Crystal Block  #
Crystal
Type Cuboid                       # Cuboid
Dimensions  12 12 45  # 45 45 80 # Dimensions of the crystal in X,Y,Z in µm
PixelsPerMicron 2                 # The computational resolution
AbsCoefCalc  RD3D                 # Tells RADDOSE-3D how to calculate the
Absorption coefficients
UnitCell   58.5 58.5 151.4    # unit cell size: a, b, c
                              # alpha, beta and gamma angles default to 90°
NumMonomers  8                # number of monomers in unit cell
NumResidues  206              # number of residues per monomer
ProteinHeavyAtoms S 17   # heavy atoms added to protein part of the monomer
SolventHeavyConc Na 1800 K 1800 S 100   # concentration of elements in the
solvent
SolventFraction 0.56        # fraction of the unit cell occupied by solvent
CALCULATEPEESCAPE TRUE
CALCSURROUNDING TRUE
SURROUNDINGHEAVYCONC Na 1800 K 1800 S 100
GONIOMETERAXIS 90

#  Beam Block  #
Beam
Type Gaussian                  # Gaussian profile beam
Flux 2.4e14
Energy 11.56                   # in keV
FWHM 2 4                       # in µm, vertical by horizontal for a Gaussian
beam
Collimation Circular 2 4

# Wedge Block #
Wedge 0 0               # Start and End rotational angle of the crystal.
                        # Start < End
ExposureTime 0.000090   # Total time for entire angular range in seconds
# AngularResolution 2   # Only change from the defaults when using very small
wedges, e.g 5°.
```

Lysozyme

```
#  Crystal Block  #
Crystal
Type Cuboid                         # Cuboid
Dimensions  5 5 5                   # Dimensions of the crystal in X,Y,Z in µm
PixelsPerMicron 10                  # The computational resolution
AbsCoefCalc  RD3D                   # Tells RADDOSE-3D how to calculate the
Absorption coefficients
UnitCell  78.6  78.6  37.8         # unit cell size: a, b, c.
                                    # alpha, beta and gamma angles default to 90°
NumMonomers  8                            # number of monomers in unit cell
NumResidues 129                           # number of residues per monomer
ProteinHeavyAtoms S 10 Na 1 Cl 3    # heavy atoms added to protein part of
the monomer
SolventHeavyConc Na 1220    # concentration of elements in the solvent
SolventFraction 0.39        # fraction of the unit cell occupied by solvent
ContainerMaterialType mixture
MaterialMixture mylar
ContainerThickness 13
CALCULATEPEESCAPE TRUE
DENSITYBASED TRUE
CALCSURROUNDING TRUE
SURROUNDINGHEAVYCONC Na 1200
GONIOMETERAXIS 90

#  Beam Block  #
Beam
Type Gaussian             # Gaussian profile beam
Flux 5.2e14
Energy 11.56              # in keV
FWHM 2 4                  # in µm, vertical by horizontal for a Gaussian beam
Collimation Circular 2 4

#  Wedge Block  #
Wedge 0 0                  # Start and End rotational angle of the crystal.
                            # Start < End
ExposureTime 0.000090      # Total time for entire angular range in seconds
# AngularResolution 2      # Only change from the defaults when using very
small wedges, e.g 5°.
```

Proteinase K

```
#  Crystal Block  #
Crystal
Type Cuboid                    # Cuboid
Dimensions  4 4 4              # Dimensions of the crystal in X,Y,Z in µm
PixelsPerMicron 10             # The computational resolution
AbsCoefCalc  RD3D              # Tells RADDOSE-3D how to calculate the
Absorption coefficients
UnitCell  68.7 68.7 109        # unit cell size: a, b, c.
                               # alpha, beta and gamma angles default to 90°
NumMonomers  8                # number of monomers in unit cell
NumResidues 279                # number of residues per monomer
ProteinHeavyAtoms S 10 Ca 2    # heavy atoms added to protein part of the
monomer
SolventHeavyConc S 120 Na 500 Ca 100 Cl 100 # concentration of elements in
the solvent
SolventFraction 0.44        # fraction of the unit cell occupied by solvent
ContainerMaterialType mixture
MaterialMixture mylar
ContainerThickness 13
CALCULATEPEESCAPE TRUE
DENSITYBASED TRUE
CALCSURROUNDING TRUE
SURROUNDINGHEAVYCONC S 120 Na 500 Ca 100 Cl 100
GONIOMETERAXIS 90

#  Beam Block  #
Beam
Type Gaussian                  # Gaussian profile beam
Flux 3.8e14
Energy 11.56                   # in keV
FWHM 2 4                       # in µm, vertical by horizontal for a Gaussian
beam
Collimation Circular 2 4

#  Wedge Block  #
Wedge 0 0               # Start and End rotational angle of the crystal.
                         # Start < End
ExposureTime 0.000090   # Total time for entire angular range in seconds
# AngularResolution 2   # Only change from the defaults when using very small
wedges, e.g 5°.
```

$A_{2A}R$

```
#  Crystal Block  #
Crystal
Type Cuboid                    # Cuboid
Dimensions  10 10 2            # Dimensions of the crystal in X,Y,Z in µm
PixelsPerMicron 10             # The computational resolution
AbsCoefCalc  RD3D              # Tells RADDOSE-3D how to calculate the
Absorption coefficients
UnitCell  40.2 179.4 142.4    # unit cell size: a, b, c.
                               # alpha, beta and gamma angles default to 90°
NumMonomers  8                # number of monomers in unit cell
NumResidues 383                # number of residues per monomer
ProteinHeavyAtoms S 22 Na 1   # heavy atoms added to protein part of the
monomer
SolventHeavyConc S 75 Na 500 Cl 150 # concentration of elements in the
solvent
SolventFraction 0.52        # fraction of the unit cell occupied by solvent
ContainerMaterialType mixture
MaterialMixture mylar
ContainerThickness 13
CALCULATEPEESCAPE TRUE
DENSITYBASED TRUE
CALCSURROUNDING TRUE
SURROUNDINGHEAVYCONC S 75 Na 500 Cl 150
GONIOMETERAXIS 90

#  Beam Block  #
Beam
Type Gaussian                  # Gaussian profile beam
Flux 3.8e14
Energy 11.56                   # in keV
FWHM 2 4                       # in µm, vertical by horizontal for a Gaussian
beam
Collimation Circular 2 4

#  Wedge Block  #
Wedge 0 0                # Start and End rotational angle of the crystal.
                          # Start < End
ExposureTime 0.000090    # Total time for entire angular range in seconds
# AngularResolution 2    # Only change from the defaults when using very
small wedges, e.g 5°.
```
